# Evaluation of the International League Against Epilepsy 1981, 1989, and 2017 classifications of seizure semiology and etiology in a population‐based cohort of children and adults with epilepsy

**DOI:** 10.1002/epi4.12562

**Published:** 2021-11-29

**Authors:** Isaac J. Egesa, Charles R. J. C. Newton, Symon M. Kariuki

**Affiliations:** ^1^ Kenya Medical Research Institute (KEMRI)‐Wellcome Trust Research Programme Kilifi Kenya; ^2^ Department of Public Health Pwani University Kilifi Kenya; ^3^ Department of Psychiatry University of Oxford Oxford UK

**Keywords:** classification, epilepsy, ILAE‐1981, ILAE‐1989, ILAE‐2017, outcomes, seizure

## Abstract

**Objective:**

The International League Against Epilepsy (ILAE) has revised the classification of epilepsies and seizures on several occasions since the original classification published in 1964. It is unclear if these changes have impacted the characterization of epilepsy, including the clinical validity of seizure semiology or epilepsy outcomes in resource‐poor areas. We aim to address this important knowledge gap.

**Methods:**

We reviewed the clinical seizure semiology and etiological data of 483 persons with epilepsy identified from a population‐based survey in rural Kenya. The seizure semiology and etiological data were classified using the 1981 (for seizures) and 1989 (for epilepsy) ILAE criteria and then reclassified according to the ILAE‐2017 criteria. Logistic regression models adjusted for potential confounders were used to measure the associations between the seizure semiology and different clinical and electroencephalographic features of epilepsy.

**Results:**

Focal (formerly localization‐related) and generalized epilepsies were lower in ILAE‐2017 (56% and 29%) than that of ILAE‐1989 (61% and 34%), *P* < .001 and *P* < .001. Combined focal and generalized epilepsy type in ILAE‐2017 accounted for 11% of epilepsies. Individual seizure types were statistically similar in both ILAE‐1981 and 2017. New classification categories in ILAE‐2017 such as unknown seizures and epilepsies were identified, and the proportions were similar to the unclassified category in ILAE‐1989, 6% and 5%, respectively. The most common causes of epilepsy were symptomatic (76%) in the ILAE‐1989 criteria, with infectious (45%) and structural (36%) causes were highest in the ILAE‐2017 criteria.

**Significance:**

Our study confirms that the two ILAE classification schemes are broadly consistent, but the introduction of the combined onset seizure category in ILAE‐2017 significantly reduces the proportion of mutually exclusive focal and generalized seizures. The comprehensive classification of etiology categories in ILAE‐2017 will facilitate appropriate treatment and improve prognosis.

**Key points**

- Combined onset focal and generalized seizures reduce the proportion of focal and generalized epilepsies in the 2017 ILAE system compared with the 1989 ILAE
- The distribution of individual seizure types does not differ between ILAE‐1981 and 2017
- Symptomatic and infectious are the common causes of epilepsy in ILAE‐1989 and ILAE‐2017, respectively

## 1 INTRODUCTION

Epilepsy is a serious neurological disorder that affects approximately 70 million people globally.^1^ It is diagnosed in people experiencing two or more unprovoked seizures occurring ≥24 hours apart, or those with one unprovoked seizure and a probability of further unprovoked seizures similar to the general recurrence risk of at least 60%.^2^ The prevalence of epilepsy in low‐ and middle‐income countries (LMIC) is two to three times higher than that in high‐income countries,^1^ with a higher proportion of symptomatic and focal epilepsy.^3^ Classification of epileptic seizures has been proposed by International League Against Epilepsy (ILAE),^4^, ^5^, ^6^, ^7^, ^8^, ^9^, ^10^ with subsequent revisions. However, there are limited data on the utility of different ILAE classifications criteria on the distribution of seizures, epilepsies, etiologies, and outcome in population‐based studies from LMIC, particularly Africa.^11^

The classification of seizure and epilepsy by the ILAE originates in 1964.^12^ Subsequent classification of seizures by ILAE‐1981 and of epilepsy and epilepsy syndrome by ILAE‐1989 has widely impacted research and clinical practice for over a decade.^5^, ^7^, ^10^ The successive versions aimed to improve classification by making it more precise, clearer, reliable, and incorporate newly identified epileptic seizures, syndromes, and etiologies.^4^ These proposals especially those published by Berg et al^4^ were influential in development of the new ILAE classification system. The newly introduced system of ILAE‐2017 classification has not been used widely, and its applicability needs to be assessed/validated, particularly in LMIC.

The advances in technology, eg, increased access to neuroimaging in urban centers of LMIC, create a need for updated information on classification of seizures, epilepsies, and etiologies. We investigated the epileptic seizure semiology and etiological classification in persons with epilepsy in a defined area in Kilifi County. We hypothesized that the proportion and distribution of seizures, epilepsies, and etiologies is similar when using ILAE‐1981, 1989, and 2017. Furthermore, we hypothesized that different epilepsy outcomes are associated with seizure types from both ILAE criteria.

## 2 MATERIAL AND METHODS

### 2.1 Study site and population

The study was conducted in Kilifi county, on the Kenyan coast, in a demographic surveillance area of 891 km^2^ (https://kemri‐wellcome.org/programme/surveillance/). The residents are mainly Mijikenda, a Bantu‐speaking community who are mainly subsistence farmers and fishermen. The area is considered one of the poorest with 55% of the population considered poor. Participants were drawn from a 2008 epilepsy survey of the general population.^13^

### 2.2 Study design and selection process

We reviewed the case records of patients (children and adults) with epilepsy identified in a 2008 survey^13^ of the general population. Recruitment of participants involved a three‐stage screening described elsewhere.^13^ Detailed clinical history, electroencephalography (EEG), and magnetic resonance imaging (MRI) were used to identify epileptic seizures, etiology, and outcomes. An EEG was recorded using a 16‐lead channel digital recording system (Grass Technologies) with electrode placement according to international 10‐20 system^14^ and reported as described in Kariuki et al.^15^ MRI was performed using a Signa HDe 1.5 Tesla, General Electric with axial TSE T2, coronal TSE T2, coronal T2 map, coronal FLAIR, e2d diff Orth ADC, and 3D FLAIR epilepsy protocols while sequences were performed with standard partition sizes (≤3 mm). Epidemiological data were also linked to Kilifi County Referral Hospital data to determine further etiology of epilepsy.

### 2.3 Review of files and data extraction

Case records were available for 936 persons who participated in the third stage of epilepsy survey, of which 483 were records of persons who met ILAE definition of epilepsy.^2^ The type of seizure semiology and underlying etiology was determined from clinical history, EEG, neuroimaging (done in 22% of the sample size), and patient's seizure description notes. Files were independently reviewed by I.E (who reviewed all files) and SK (who randomly reviewed 30% of files). Of the 483 files reviewed (either by one or both reviewers), inconsistency or unclear details in seizure phenotypes or features were documented in 5% and these were resolved through consensus in consultation with CN who is a pediatric neurologist. The data extracted included age, marital status, education level, employment status, EEG findings, neuroimaging findings, epilepsy outcomes, epilepsy classification, and etiology according to ILAE‐1981, 1989, and 2017. Data were extracted into Excel (Microsoft) spreadsheet before being transferred into the analysis software.

### 2.4 Definition of terms

Epilepsy and seizure phenotypes (Table 1) were based on ILAE recommendations of 1981, 1989,^5^, ^7^, ^16^ and 2017.^8^, ^9^ Epilepsy was defined according to the recommendation by ILAE commission on epidemiology as ≥2 unprovoked seizures occurring 24 hours apart, with at least one seizure in the last 12 months.^17^ Status epilepticus was defined as a history of seizures lasting for 30 minutes or more with impaired consciousness or intermittent seizures lasting for 30 minutes or more without regaining consciousness.^18^

**TABLE 1:** Comparison between the ILAE‐1981, 1989 and 2017 seizures and epilepsies

**1981 seizure classification**			**2017 seizure classification**			
**Partial seizures** **Simple partial** *Motor signs Somatosensory* *Autonomic* *Psychic* **Complex partial** *Automatism* *Aura*	**Generalized seizures** *Absence* *Atypical absence* *Myoclonic* *Clonic* *Tonic* *Tonic‐clonic* *Atonic* *others*	**Unclassified seizures**	**Focal seizures** **Aware** **Motor onset** *Automatism, atonic,* *clonic, epileptic spasm* *Hyperkinetic, tonic,* *myoclonic* **Nonmotor** *Autonomic, emotional* *Behavior arrest* *sensory* **Impaired awareness** **Motor onset** *Automatism, atonic,* *Clonic, epileptic spasm* *Hyperkinetic, tonic,* *Myoclonic* **Nonmotor** *Autonomic, emotional* *Behavior arrest* *sensory*	**Generalized seizures** **Motor onset** *Tonic‐clonic* *Clonic, tonic,* *Myoclonic* *Myoclonic‐tonic‐clonic,* *atonic,* *Epileptic spasms* **Nonmotor** *Typical absence,* *Atypical absence,* *Myoclonic,* *Eyelid myoclonia*	**Unknown seizures** **Motor** *Tonic‐clonic* *Epileptic spasms* **Nonmotor** *Behaviour arrest* Unclassified	
Partial evolving to secondarily generalized seizures			Focal to bilateral tonic‐clonic seizure			
**1989 epilepsy classification**			**2017 epilepsy classification**			
Localization‐related (Focal, local, partial)	Generalized	Undetermined	Focal	Generalized	Combined focal and generalized	Unknown
**1989 etiology classification**			**2017 etiology classification**			
Symptomatic, idiopathic, cryptogenic			Structural, infectious, immunological, metabolic, genetic			

Seizure classification according to ILAE‐1981 vs. ILAE‐2017, epilepsy classification according to ILAE‐1989 vs. ILAE‐2017 and aetiology classification according to ILAE‐1989 vs. ILAE‐2017

Epilepsy age at onset was defined as age at first unprovoked seizure.^17^ Neurological deficit and learning abilities were defined based on the assessment of cognitive impairment of participant's awareness of person, place, and time and ability to follow standardized instructions during neurological examination.^3^ We determined the marriage and employment status based on adults (>18 years).^3^

### 2.5 Classification of seizures

The seizure semiology was classified according to ILAE‐1981^10^, ^16^ and ILAE‐2017.^9^ In ILAE‐1981, the seizures were classified into partial, generalized, and unclassifiable seizure onset. The partial seizures involved initial clinical and EEG features limited to one cerebral hemisphere while generalized seizures involved initial clinical and EEG abnormalities in both hemispheres. The partial seizures were further classified into simple partial (retention of conscious state), complex partial (impaired conscious state), and partial seizures evolving to secondarily generalized seizures,^10^, ^16^ (Table 1).

In ILAE‐2017, an epileptic seizure was classified into focal, generalized, and unknown seizure onset. The focal onset seizures were defined as those beginning within networks confined to one hemisphere while generalized onset involved seizures arising at some point within, and immediately involving bilaterally distributed networks. Focal onset was then categorized based on the level of awareness (knowledge of self and environment) into focal aware, focal impaired awareness, and focal to bilateral tonic‐clonic seizures. A further level of classification was based on motor and nonmotor onset^9^ (Table 1).

### 2.6 Classification of epilepsies

Epilepsies were classified according to ILAE‐1989^7^ and ILAE‐2017.^8^ In ILAE‐1989, epilepsies were categorized into localization‐related (focal, local, or partial), generalized, and undetermined. The localized‐related (focal, local, or partial) epilepsy referred to epilepsies whose seizure semiology or EEG and neuroimaging findings indicated localized origin. On the other hand, generalized epilepsies included initial seizure semiology involving both hemispheres. Undetermined epilepsy included persons with no evidence of either focal or generalized seizure onset.^7^

In ILAE‐2017, epilepsies were classified into focal, generalized, combined generalized and focal, and unknown epilepsies. Focal epilepsy was defined as the lateralized semiology and/or epileptiform discharges on EEG, whereas generalized epilepsy was defined as the bilateral seizure semiology and/or epileptiform discharges on the EEG. The combined generalized and focal epilepsies involved persons who had both focal and generalized seizures. The unknown category of epilepsy was based on persons with epilepsy diagnosis with insufficient information to classify as either focal or generalized epilepsy.^8^

### 2.7 Classification of epilepsy etiology

Etiology in ILAE‐1989 was classified as symptomatic (known causes), idiopathic (no underlying cause with a presumed hereditary predisposition), or cryptogenic (hidden or unknown cause).^7^ Etiology in ILAE‐2017 was classified as structural, infectious, possible genetic, or undetermined causes.^8^ The immunologic causes of ILAE‐2017 were classified as undetermined causes in this setting due to limited technology. We identified these ILAE etiologies using EEG findings and MRI, which was done for people with focal epilepsy and a selected proportion of those with generalized epilepsy. Participants with adverse prenatal events (newborn unable to breathe, cry, or breastfeed immediately after birth for seizure onset <18 years of age) and high parasite antibodies titer for exposure to neuroinfections were considered as potential causes of epilepsy.^13^ Additional information on seizure semiology, age at onset, and previously identified risk factors and proximate causes of epilepsy used in this analysis was previously described.^3^, ^19^

In this study, we were interested in examining the association of seizure semiology under the ILAE schemes of 1981, 1989, and 2017 with outcomes such as poor marriage prospects, lack of schooling, intellectual disability, convulsive status epilepticus, and neurological deficits.

### 2.8 Statistical analysis

Statistical analyses were performed using the stata version 15 statistical software (Stata Corporation). Age of participants and age at seizure onset were stratified according to prior determined age bands for investigating differences in distribution of seizures and epilepsies of ILAE‐1981, 1989, and 2017 classification system. The degree of association between ILAE‐1981, 1989, and 2017 epileptic seizures and different epilepsy outcomes was measured using logistic regression. A variable with a *P*‐value <.25 was used to build a multivariable model in a stepwise regression technique. Pearson's chi‐squared test and Fisher's exact test (where observations were infrequent) were used to compare categorical variables. Comparison of continuous variables was done using student's *t*‐test or Mann‐Whitney *U* test (in nonparametric distribution).

### 2.9 Ethical approval

Permission was approved by the local Institutional Ethical Committee and the National Ethics Review Committee of Kenya. Informed consent was obtained from adults' participants and assent from children below 18 years.

## 3 RESULTS

### 3.1 Demographics

The study had 256/483 (53.0%) children. Male represented 45.4% of the study sample, and 48.4% of the male were children. The median age of participants was 17 years (interquartile range [IQR] 9‐27). The median age at seizure onset was 3 years (IQR 1.0‐11.5), with no significant differences between the sexes (*P* = .768). Seventy‐two percent of children had onset of seizure under the age of 5 years with a median of 1.6 years (IQR 0.5‐4.3). The distribution of other general characteristics of persons with epilepsy is described in Table 2.

**TABLE 2:** General characteristics of persons with epilepsy

Participants N = 483	Total N = 483	Children N = 256	Adults N = 227	*P*‐value	Male N = 219	Female N = 264	*P*‐value
Median age years (IQR)	17 (9‐27)	10 (6‐14)	28 (22‐43)	<.0001	18 (9.0‐26.0)	16 (10.0‐27.5)	.863
Male (N, %)	219 (45.4)	114 (43.2)	150 (56.8)	.065	—	—	
Age onset, median years (IQR)	3.0 (1.0‐11.5)	1.6 (0.5‐4.3)	10.0 (2.3‐25.4)	<.0001	3.5 (1.0‐13.0)	2.6 (0.9‐10.0)	.768
Unmarried adults (N, %)	133 (27.5)		133 (100.0)		70 (52.6)	63 (47.4)	.241
Unschooled (N, %)	234 (47.6)	132 (52.4)	102 (45.3)	.124	120 (55.8)	114 (43.5)	.007
Abnormal EEG (N, %)	203 (42.0)	122 (60.1)	81 (39.9)	.004	88 (43.3)	115 (56.7)	.990
Abnormal MRI (N, %)	61 (12.6)	31 (50.8)	30 (49.2)	.059	28 (45.9)	33 (54.1)	.392
Neurological deficits (N, %)	94 (19.7%)	53 (56.4)	41 (43.6)	.441	36 (38.3)	58 (61.7)	.141
Learning difficulties (N, %)	141(29.2%)	78 (55.3)	63 (44.7)	.481	56 (39.7)	85 (60.3)	.128
Burns (N, %)	104 (21.8)	42 (40.4)	62 (59.6)	.004	60 (57.7)	44 (42.3)	.003
Status epilepticus (N, %)	165 (34.1)	103 (62.4)	62 (37.6)	.002	78 (42.3)	87 (52.7)	.459

### 3.2 Distribution of seizures classification

Partial onset seizures were more common than generalized onset seizures in ILAE‐1981 classification (294 [69.9%] vs 162 [33.5%]; *P* < .001). Similarily, focal onset seizures were more frequent than generalized seizures in ILAE‐2017 classification (299 [61.9%] vs 157 [32.5%]; *P* < .001) (Table 3).

**TABLE 3:** Distribution of seizures and epilepsy types according to ILAE‐1981, 1989 and 2017 among childen and adult population

	Frequency N = 483 (%)		Frequency N = 483 (%)
**ILAE‐1981 seizures types**		**ILAE‐2017 seizure type**	
Partial	294 (60.9)	Focal	299 (61.9)
Generalized	162 (33.5)	Generalized	157 (32.5)
Unclassified	27 (5.6)	Unknown	27 (5.6)
**ILAE‐1989 epilepsy types**		**ILAE‐2017 epilepsy types**	
Localized‐related	294 (60.9)	Focal	268 (55.5)
Generalized	162 (33.5)	Generalized	139 (28.8)
		Combined	53 (10.9)
Undetermined	27 (5.6)	Unknown	23 (4.8)

Seizure subtypes ILAE 1981	Children n (%)	Adults n (%)	*P*‐value	Seizure subtypes ILAE2017	Children n (%)	Adults n (%)	*P*‐value
Generalized tonic‐clonic	31 (44.9)	38 (55.1)	.147	Generalized tonic‐clonic	31 (44.9)	38 (55.1)	.147
Simple partial	54 (47.8)	59 (52.2)	.204	Focal aware	42 (46.7)	48 (43.3)	.182
Complex partial	57 (62.0)	35 (38.0)	.056	Focal impaired aware	57 (62.0)	35 (38.0)	.056
Partial to secondarily generalized	42 (47.2)	47 (52.8)	.224	Focal to bilateral tonic‐clonic	42 (47.2)	47 (52.8)	.224

**FIGURE 1:**
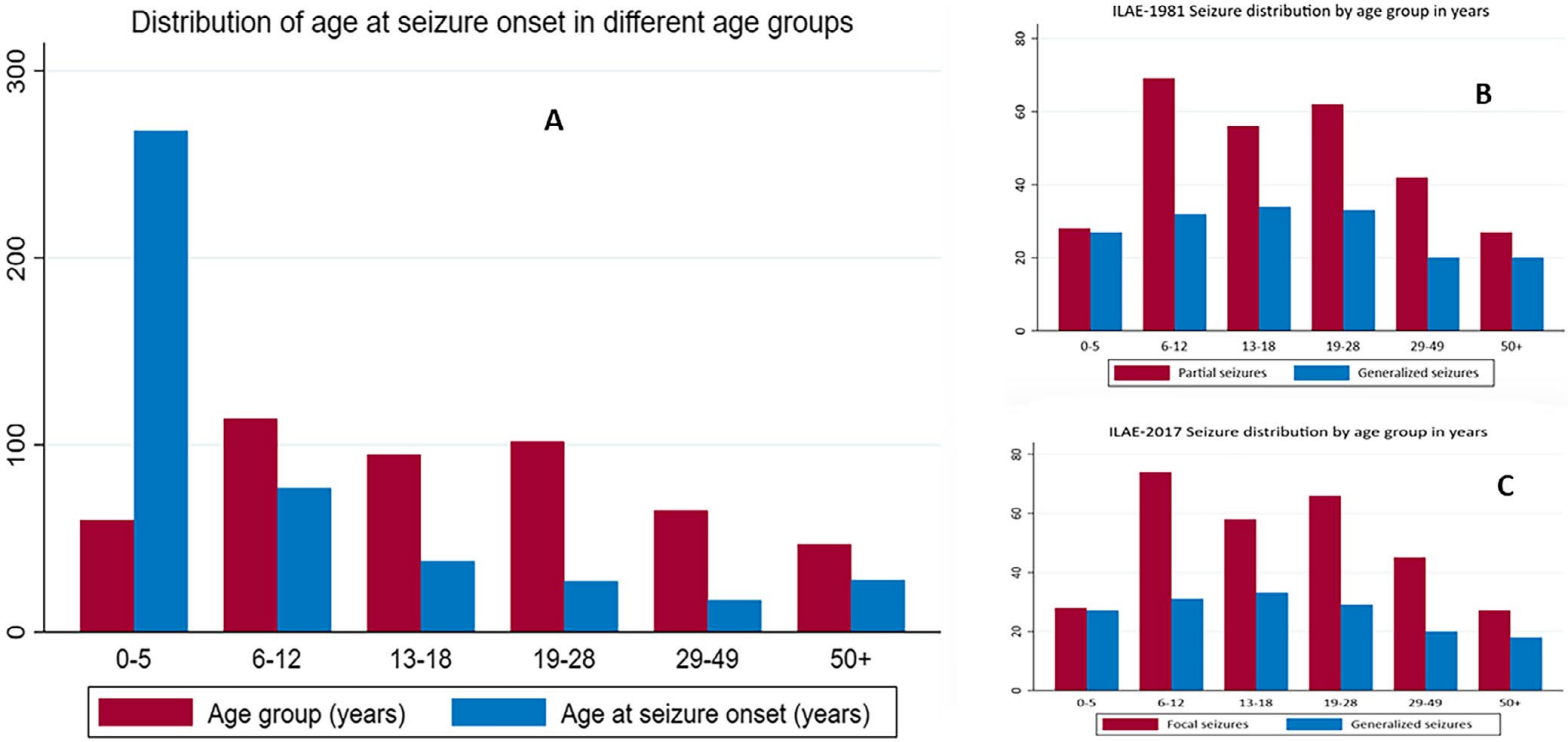
Distribution of seizures and age at onset among age groups. (A) age at the onset of seizures, (B) distribution of focal and generalized seizures according to ILAE‐1981, (C) distribution of focal and generalized seizures according to ILAE‐2017”

Half of the persons with epilepsy had seizure onset at the age of 5 years and below. Focal seizures were the most frequent seizure type in all age at onset categories, across both ILAE‐1981 and ILAE‐2017 classifications (Figure 1). Earlier onset of seizures was associated status epilepticus (*P* < .001), learning difficulties (*P* < .001), with not attending school (*P* < .001), and mortality (*P* = .003).

The distribution of specific seizure types such as tonic, myoclonic, atonic, clonic, tonic‐clonic, and absence was similar across the two ILAE classification systems. The distribution of seizure types for ILAE‐1981 and ILAE‐2017 did not differ between children and adults (Table 3). There was no difference in the distribution of focal and generalized seizures among age groups for both ILAE‐1981 and 2017 classification schemes (Figure 1).

### 3.3 Distribution of epilepsy classification

We reported 294 (60.9%) localized‐related (focal, local, and partial) vs 162 (33.5%) generalized epilepsy and 268 (55.5%) focal vs 139 (28.8%) generalized epilepsy for ILAE‐1989 and 2017, respectively (Table 3). The introduction of combined generalized and focal epilepsy in the ILAE‐2017 substantially reduced the proportion of focal onset, from 60.9% to 55.5% (*P* < .001) for mutually exclusive focal epilepsies, and the proportion with generalized from 33.5% to 28.8% (*P* < .001). There was a similar distribution for the proportion of undetermined onset epilepsies in ILAE‐1989 and unknown onset in ILAE‐2017 was similar (4.8% vs 5.6%, respectively) (Table 3).

### 3.4 Etiology of epilepsy

In ILAE‐1989, symptomatic was the commonest cause of epilepsy and was higher in children (92.4%) than that in adults (76.2%); *P* < .001, (Table 4). Idiopathic and cryptogenic causes of epilepsy were reported in 18.6% and 8.2%, respectively. Under ILAE‐2017 classification, the common etiological category was infectious (44.8%) and structural (36.4%) causes. Structural causes were higher in children (44.9%) than that in adults (26.9%), *P* < .001. About 24.6% of persons had undetermined epilepsy causes. The proportion of identifiable causes of epilepsy was similar in the two classification systems (Table 4). Structural causes and infectious causes were associated with early onset of epilepsy (*P* = .004 and *P* = .037, respectively). The other causes of epilepsy in ILAE‐2017 did not show an association with the age at the onset of epilepsy.

**TABLE 4:** Aetiological classification of epilepsy in ILAE‐1989 and 2017 in epilepsy

Etiology category	Children N = 256 (%)	Adults N = 227 (%)	Total N = 483 (%)	*P*‐value
1989 etiology				
Symptomatic	196 (92.4)	173 (76.2)	369	<.001
Idiopathic	40 (18.9)	34 (18.18)	74 (18.6)	.860
Cryptogenic	20 (7.8)	20 (8.8)	40 (8.2)	.462
Total	256	227	483	
2017 etiology				
Infectious	111 (43.3)	104 (45.8)	215 (44.5)	.588
Structural	115 (44.9)	61 (26.9)	176 (36.4)	<.001
Undetermined			119 (24.6)	
Total	256	227	483	

Epilepsy aetiology identified by electroencephalography (EEG) findings, magnetic resonance imaging (MRI) findings, prenatal events, high parasite antibody titres for exposure to neuro‐infection, and previously identified risk factors and proximate causes of epilepsy.^3^, ^19^

Symptomatic causes of epilepsy in ILAE‐1989 were correlated with structural causes of ILAE‐2017 (Rho 0.35, *P* = .050), but infectious causes were not correlated (Rho −0.32, *P* = .999). The ILAE‐2017 classification of epilepsy etiology allowed for the use of more than one etiological category in the same patient. The possible metabolic, genetic, and immunological causes of epilepsy in ILAE‐2017 were correlated with structural etiology in ILAE‐1989 (*P* < .001).

Abnormal EEG was reported in 42.0% of persons with epilepsy and was defined as focal in 40.4%, and the remainder was generalized abnormalities (Table 2). There was no significant correlation for partial seizure semiology vs focal EEG (*P* = .45 for ILAE‐1981 and *P* = .34 for ILAE‐2017) nor there was a significant correlation for generalized seizure semiology vs generalized EEG (*P* = .50 for ILAE‐1981 and *P* = .36 for ILAE‐2017) (Table 5).

**TABLE 5:** Multivariate analysis for the association between seizure semiology and epilepsy outcome

Outcomes	Status epilepticus OR (95% CI)	Unmarried OR (95% CI)	Unschooled OR (95% CI)	Mortality OR (95% CI)	Learning difficulties OR (95% CI)	Neurology difficulties OR (95% CI)	Focal EEG OR (95% CI)	Generalized EEG OR (95% CI)
Seizure types								
Focal seizures ILAE‐1981								
Simple partial	1.38 (0.61‐3.14)	1.16 (0.55‐2.46)	1.48 (0.71‐3.10)	0.39 (0.10‐1.49)	2.59 (0.84‐8.03)	1.11 (0.43‐2.85)	0.79 (0.39‐1.61)	
Complex partial	2.18 (0.95‐5.00)	0.78 (0.36‐1.71)	1.68 (0.78‐3.57)	0.60 (0.15‐2.31)	6.00^a^ (1.96‐18.3)	1.56 (0.61‐4.04)	1.67 (0.86‐3.28)	
sGTC	1.91 (0.82‐4.42)	1.90 (0.88‐4.13)	1.51 (0.70‐3.25)	0.99 (0.28‐3.52)	5.88^a^ (1.91‐18.1)	1.18 (0.44‐3.13)	1.29 (0.64‐2.59)	
Generalized seizures ILAE‐1981								
Tonic‐clonic	0.95 (0.38‐2.36)	0.93 (0.41‐2.11)	2.26 (1.01‐5.01)	0.69 (0.18‐2.70)	4.64^a^ (1.46‐14.75)	0.73 (0.25‐2.16)		1.13^a^ (0.55‐2.30)
Clonic	1.57 (0.57‐4.31)	0.87 (0.33‐2.31)	1.53 (0.60‐5.00)	0.23 (0.02‐2.25)	4.38^a^ (1.24‐15.49)	0.98 (0.29‐3.27)		1.68 (0.714‐3.95)
Tonic	1.00 (0.33‐3.01)	0.43 (0.13‐1.38)	1.85 (0.68‐5.10)	1.24 (0.18‐8.40)	3.00 (0.78‐11.58)	1.01 (0.28‐3.61)		1.39 (0.53‐3.65)
Myoclonic	2.35 (0.48‐11.5)	2.17 (0.49‐9.51)	1.18 (0.27‐5.10)	0.81 (0.07‐9.47)	2.65 (0.40‐17.52)	4.08 (0.86‐19.41)		1.92 (0.37‐9.90)
Absence	0.47 (0.05‐4.58)	0.77 (0.12‐4.82)	1.35 (0.23‐7.62)	—	5.06 (0.68‐37.38)	2.76 (0.41‐18.4)		3.71 (0.33‐42.1)
Focal seizures ILAE 2017								
Focal aware	1.37 (0.70‐2.67)	1.54 (0.60‐3.99)	1.18 (0.48‐2.88)	1.31 (0.14‐12.2)	6.06 (0.76‐48.21)	1.33 (0.35‐5.13)	0.99 (0.47‐2.08)	
Focal impaired aware	2.43^a^ (1.28‐4.64)	1.06 (0.41‐2.75)	1.57 (0.64‐3.84)	1.46 (0.16‐13.5)	16.27^a^ (2.10‐126.1)	2.36 (0.64‐8.70)	1.78 (0.90‐3.50)	
FBTC	2.13^a^ (1.11‐4.12)	2.58^a^ (0.99‐6.70)	1.42 (0.57‐3.50)	2.43 (0.27‐21.6)	16.05^a^ (2.06‐125.2)	1.79 (0.48‐6.75)	1.37 (0.68‐2.77)	
Focal motor	2.50^a^ (1.00‐6.24)	1.82 (0.58‐5.70)	2.18 (0.72‐6.58)	0.49 (0.02‐8.94)	9.19^a^ (1.03‐81.58)	3.16 (0.73‐13.7)	0.69 (0.18‐2.58)	
Generalized seizures ILAE 2017								
Tonic	1.32 (0.38‐4.70)	0.58 (0.16‐2.11)	1.74 (0.58‐5.23)	3.06 (0.22‐42.1)	8.16 (0.91‐72.47)	1.53 (0.32‐7.26)		1.35 (052‐3.51)
Tonic‐clonic	1.27 (0.43‐3.82)	1.26 (0.47‐3.38)	2.12 (0.84‐5.36)	1.70 (0.18‐15.8)	12.62^a^ (1.59‐100.0)	1.11 (0.27‐4.53)		1.04 (0.52‐3.51)
Atonic	1.60 (0.30‐8.45)	2.06 (0.47‐9.05)	0.95 (0.22‐4.18)	3.37 (0.24‐46.9)	9.56 (0.85‐106.9)	2.57 (0.42‐15.8)		—
Clonic	2.09 (0.64‐6.87)	1.18 (0.38‐3.62)	1.44 (0.51‐4.10)	0.56 (0.03‐10.1)	11.93 (1.41‐100.7)	1.48 0.32‐6.69)		1.62 (0.69‐3.78)
Nonmotor	1.71 (0.40‐7.34)	1.94 (0.52‐7.32)	1.17 (0.32‐4.23)	1.39 (0.07‐26.7)	9.36 (0.93‐94.24)	5.28^a^ (1.07‐26.0)		—

sGTC‐secondarily generalized tonic‐clonic, FBTC‐focal to bilateral tonic‐clonic, OR = Odds Ratio, CI = Confidence Interval, a = Significant level (set at p = 0.05) otherwise not significant. Association between seizure types of ILAE‐1981 and 2017 with different epilepsy outcomes. The Significance use of superscript 'a' is at 0.05

### 3.5 Association between seizure semiology and epilepsy outcomes

There was considerable difference in the proportion of persons with epilepsy with burns among children 16.7% vs adults 27.6% (*P* = .004) and male 27.9% vs female 16.3% (*P* = .003), and status epilepticus among children 41.7% vs adults 28.2% (*P* = .002). More than one third (38.1%) of persons with epilepsy were not married without significant differences between the sexes (*P* = .059).

Status epilepticus was twofold likely to occur in focal impaired awareness seizures (OR 2.43, *P* = .007), focal to bilateral tonic‐clonic seizures (OR 2.43, *P* = .023), and focal motor seizures (OR 2.50, *P* = .050). Neither generalized seizures of ILAE‐1981 nor of ILAE‐2017 was significantly associated with status epilepticus.

Learning difficulties were associated with complex partial seizures (OR 6.00, *P* = .002) of ILAE‐1981 and focal to bilateral tonic‐clonic seizures (OR 16.05, *P* = .008) of ILAE‐2017 (Table 5). Inability to get married was significantly associated with focal to bilateral tonic‐clonic seizure using ILAE‐2017 (OR 2.58; *P* = .051) but reported an OR of 1.91 (95%CI 0.82‐4.42) for secondarily generalized tonic‐clonic using ILAE‐1981. Neither neurological deficits nor mortality was associated with seizure semiology in both ILAE‐1981 and 2017 systems of classification.

## 4 DISCUSSION

Findings of this study showed a reduction in the proportion of focal and generalized epilepsies in the 2017 ILAE system compared with the 1989 ILAE system. The category of combined onset of seizures in ILAE‐2017 explains the significant difference observed. The distribution of individual seizure types did not differ between ILAE‐1981 and ILAE‐2017, perhaps because only terminologies changed. Similar to other epilepsy studies,^20^, ^21^, ^22^ focal seizures were the most common in all age groups, for both classification systems, asserting that most epilepsy in this area is symptomatic or has identifiable causes, but underlying causes can be identifiable, eg, through neuroimaging.^3^ More than half of persons with epilepsy had a seizure onset at the age of 5 years and below, a period when many risk factors for epilepsy, eg, malaria, are common. Structural causes under the ILAE‐2017 system were less frequent than symptomatic causes in ILAE‐1989 system, probably because some were accommodated as infectious causes in ILAE‐2017. Focal seizure semiology was associated with status epilepticus, learning difficulties, and poor marriage prospects, as previously documented.^3^

### 4.1 Distribution of epileptic seizure and epilepsy types

The introduction of a combined epilepsy phenotype (focal and generalized), which accounted for 11% of patients, reducing proportion of focal and generalized epilepsy in the 2017 scheme, should be considered in future pathogenetic studies including genetics. Epileptic seizures that were unclassified in ILAE‐1981 and 1989 could finally be categorized into unknown onset seizures, which is relatable, and applicable in resource‐limited areas where access to diagnostic facilities such as EEG and neuroimaging is poor.^8^

The number of people diagnosed with specific individual seizure subtypes or even those difficult to classify was largely similar between the two classification systems. Therefore, this aids in the interpretation and comparison of related outcomes based on epilepsy phenotypes from recent and previous ILAE classification systems. Despite the identified merits of the ILAE‐2017 classification, limitations such as the use of complicated redundant terminology, lack of detailed information on seizure evolution, and inadequate guidelines to identify seizure semiology have emerged.^23^

### 4.2 Etiology of epilepsy

Causes of epilepsy are common in early childhood, when onset of epilepsy for most participants was unsurprisingly reported (84%) (<18 years).^24^ The proportions of symptomatic (76%) or structural (77%) epilepsies are higher than those previously identified^25^; defining more than one etiology in a patient could explain this small difference.^8^ An infectious etiology (44%) and structural etiology (36%) were the most common using the ILAE‐2017 classification; these are the most common causes of epilepsy globally and in LMIC.^26^, ^27^ Infectious etiology may lead to brain damage, eg, viral encephalitis, which can be classified as acquired structural etiological epilepsy.^8^

With the ILAE‐1989 etiological classification, 37% of persons with epilepsy had no identifiable (unknown) causes of epilepsy (idiopathic and cryptogenic), while the proportion fell off to 5% with unknown etiology in the ILAE‐2017. However, the proportion of unidentifiable (unknown) causes (23%) in ILAE‐1989 was similar to combined proportions of presumed genetics, immunological, metabolic, and unknown causes (23%) in ILAE‐2017. With availability of infrastructure eg, assays and genetic screening, the new scheme of classification has a higher specificity, unlike the previous ones, which lumped different etiology together.

Symptomatic (ILAE‐1989) etiological causes were defined more in children than adults (*P* < .001); children could be sensitive to early life insults from infections and birth injury. Structural etiology (ILAE‐2017) shows a similar trend distribution among children and adults (*P* < .001), as neuroimaging helped to reveal underlying abnormalities in both groups.

### 4.3 Association between seizure semiology and epilepsy outcome

Status epilepticus and intellectual disability were common in focal unaware seizure of ILAE‐2017 (complex partial seizure in ILAE‐1981), and it is thought this was due to the reverberating seizure activity in hippocampal and parahippocampal structures.^28^, ^29^ Learning difficulties were associated with focal seizures in both schemes, and these focal seizures are known to originate from frontal and temporal lobe (within the mesial structures)^30^, ^31^ that affect memory, learning, and executive function.^32^

Focal to bilateral tonic‐clonic seizure of ILAE‐2017 (formerly, partial evolving to secondarily generalized seizures ILAE‐1981) was significantly associated with poor marriage prospects, probably explained by the stigma associated with the dramatic nature of convulsive epilepsies. Seizure semiology of ILAE‐1981, 1989, and 2017 was not associated with mortality, suggesting other nonseizure factors; causes of death such as sudden unexplained death in epilepsy (SUDEP), burns, or drowning should be explored.^33^, ^34^ SUDEP is common in generalized epilepsy^35^ and need to be defined and characterized in LMIC settings.

### 4.4 Strengths and limitations of the study

The use of standard ILAE definitions makes the comparison of classifications of epilepsy easier and consistent universally. Population‐based studies like ours are representative and easily generalizable in LMIC settings. Comprehensive investigations such as EEG, MRI, and clinical examination by epilepsy specialists were used in identifying etiology and classification.

However, our findings are from LMIC setting that cannot be generalized to the worldwide epilepsy population. Specifically, the underlying causes, and the proportion of symptomatic and structural epilepsy, thereof, may be different between our setting and those from high‐income countries. Lack of assays to define metabolic epilepsy and genetic screening limited exploration of epilepsy etiology in this setting. Also, exposure to infections was based on cross‐sectional assays and does not necessarily imply causality, and there was limited number of studies comparing the utility of the former and latter ILAE classification in different settings.

## 5 CONCLUSIONS

The study provides insight into the applicability of the new ILAE classification in LMIC. The new scheme of classification introduces the combined focal and generalized epilepsy, which significantly refines the number of people diagnosed with focal and generalized epilepsy. The unknown onset epilepsies and unknown etiology make the new scheme of classification easier to implement. Multiple etiology categories and classification of seizures based on onset and level of awareness make this classification clearer in informing appropriate treatment and improving prognosis. Understanding epilepsy outcomes that are consistent with specific seizure semiology informs preventive measures to improve epilepsy prognosis.

## CONFLICT OF INTEREST

No competing interest were disclosed. All the authors have agreed for the authorship, read and approved the final version of the manuscript. We confirm that we have read the Journal position on issues involved in ethical publication and affirm that this report is consistent with those guidelines.
